# Hypereosinophilia: clinical and therapeutic approach in 2025

**DOI:** 10.1097/ACI.0000000000001078

**Published:** 2025-05-21

**Authors:** Giusi Taurisano, Maria Clara Ruffi, Silvia Canalis, Giulia Anna Maria Luigia Costanzo

**Affiliations:** Department of Medical Sciences and Public Health, University of Cagliari, Cagliari, Italy

**Keywords:** classification, eosinophils, hypereosinophilia, hypereosinophilic syndrome, treatment

## Abstract

**Purpose of review:**

This review addresses the clinical and biological complexities of hypereosinophilia (HE) and hypereosinophilic syndrome (HES), highlighting the need for improved diagnostic frameworks and therapeutic strategies. Due to the increasing recognition of HE and its potential for severe multiorgan involvement, a structured, multidisciplinary approach to diagnosis and management is essential for optimizing patient outcomes.

**Recent findings:**

Recent literature categorizes HE into hereditary, reactive, and neoplastic forms, with significant advancements in defining associated conditions and their pathophysiological mechanisms. Clinical manifestations range from asymptomatic eosinophilia to life-threatening complications involving the skin, lungs, gastrointestinal tract, heart, and nervous system. Corticosteroids remain the first-line treatment across most subtypes. Imatinib has shown high efficacy, particularly in patients with FIP1L1::PDGFRA fusion. However, therapeutic resistance and relapse still occur. Biologic therapies targeting interleukin (IL)-5 or its receptor, such as mepolizumab and benralizumab, have demonstrated promise in reducing eosinophils counts and preventing flare-ups. Additional agents under investigation include dupilumab and lirentelimab.

**Summary:**

The findings highlight the importance of accurate classification and tailored management of HE and HES, which are crucial for preventing organ damage and improving quality of life. Ongoing clinical trials and research will expand therapeutic options, clarify underlying mechanisms, and address current unmet needs.

## INTRODUCTION

**FIGURE 1:**
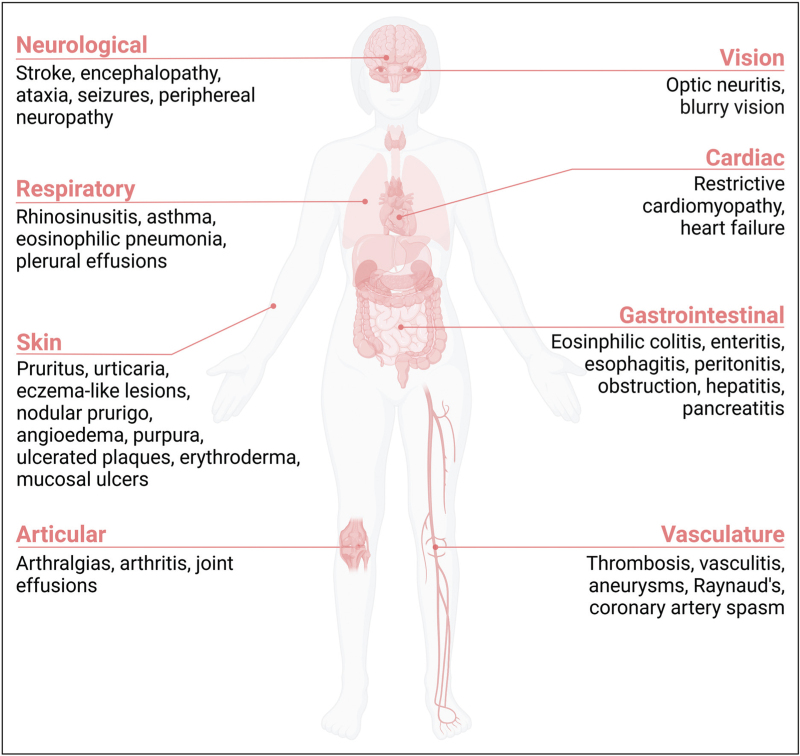
Clinical manifestations of HES. Imagine created by Biorender.

Eosinophilia is defined as an increased eosinophil count in peripheral blood, exceeding the normal range of 0.35–0.5 × 10^9^/l (3–6% of total leukocytes) [1^▪^,^2^^▪▪^,3^▪^]. Based on absolute eosinophil count (AEC), it is classified into mild (0.5–1.49 × 10^9^/l), moderate (1.5–5.0 × 10^9^/l) and severe (>5.0 × 10^9^/l) [2^▪▪^]. Hypereosinophilia (HE) is defined as an elevation in AEC exceeding 1.5 × 10^9^/L [2^▪▪^]. As stated by the 2021 Working Conference on Eosinophil Disorders and Syndromes, HE is considered persistent when this threshold is recorded on two separate occasions at least two weeks apart [3^▪^]. When HE is associated with organ damage and no alternative explanation is identified, the condition is defined as Hypereosinophilic Syndrome (HES) [1^▪^,^4^]. According to the ICOG-EO criteria, the diagnosis of HES requires: (i) the presence of blood and/or tissue HE, (ii) HE-associated organ damage, and (iii) exclusion of other underlying disorders or pathology as the primary cause [3^▪^,^4^,^5^]. Tissue HE is defined by one or more of the following criteria: (i) eosinophils constitute more than 20% of all nucleated cells in bone marrow sections, (ii) a pathologist identifies extensive (massive) eosinophil infiltration in tissue beyond normal physiological ranges, or (iii) immunostaining reveals significant extracellular deposition of eosinophil granule proteins, such as eosinophil major basic protein (eMBP1) or eosinophil peroxidase (EPX) [1^▪^,3^▪^,^4^]. Valent *et al.* highlight that tissue HE may occur without peripheral blood (PB) HE; however mild blood eosinophilia is usually observed [3^▪^]. Originally, the definition of HES required both PB HE and HE-related organ damage, regardless of tissue HE [4,3^▪^]. However, tissue HE with organ damage can occur without PB HE, typically involving a single organ—termed “tissue-restricted” or “organ-restricted (mono-organ) HES” [3^▪^] (see Table 1 for a schematic comparison of eosinophilia, HE, tissue HE, and HES, including the tissue-restricted subtype) (Fig. 1) 

**Box 1:**
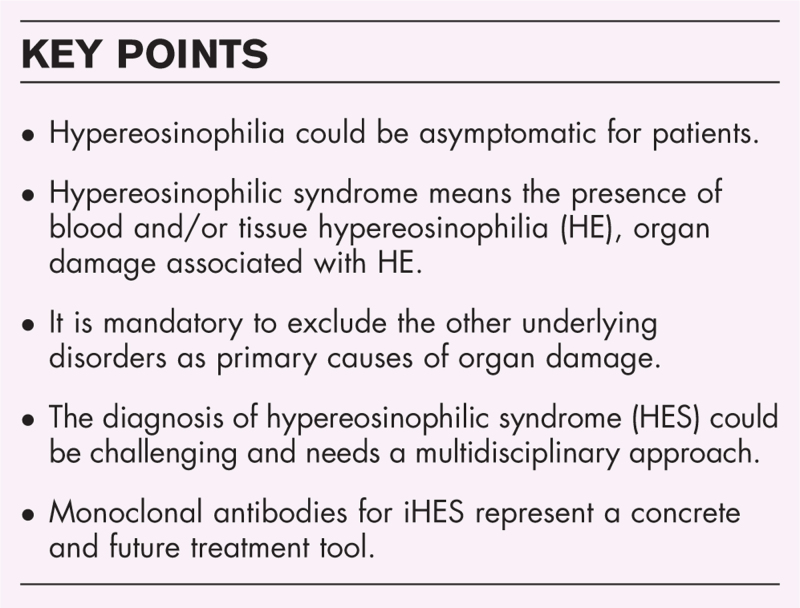
no caption available

**Table 1:** Distinction between eosinophilia, hypereosinophilia (HE), tissue HE, and hypereosinophilic syndrome (HES), including the mono-organ/tissue-restricted variant

Entity	Definition	Criteria	Notes
Eosinophilia	Elevated eosinophil count in peripheral blood	AEC > 0.5 × 10^9^/l	Classified as: mild (0.5–1.49) moderate (1.5–5.0) severe (>5.0 × 10^9^/l)
Hypereosinophilia (HE)	Marked elevation of eosinophils	AEC > 1.5 × 10^9^/l Persistent: AEC > 1.5 × 10^9^/l on ≥2 occasions, ≥2 weeks apart	May or may not be associated with tissue infiltration or symptoms
Hypereosinophilic syndrome (HES)	HE associated with organ damage, excluding secondary/reactive causes	All of the following: Blood and/or tissue HE HE-related organ damage Exclusion of other causes	Includes classic PB HE + damage and tissue-restricted (mono-organ) forms
Tissue HE	Excessive eosinophils in tissue	≥1 of the following: >20% eosinophils in BM Pathologist-documented massive infiltration Granule protein deposition (eMBP1/EPX)	May occur with or without (in rarer cases) peripheral blood HE
Tissue-restricted (mono-organ) HES	Organ-specific damage due to eosinophilic infiltration, without peripheral blood HE	Tissue HE with documented organ damage, in the absence of PB HE	Typically involves a single organ; may present diagnostic challenge in the absence of blood HE

This classification integrates peripheral blood eosinophil counts, tissue findings and clinical manifestations. Definitions are based on current consensus criteria (e.g., ICOG-EO, 2021 Working Conference; WHO 2024). AEC, absolute eosinophil count; HE, hypereosinophilia; HES, hypereosinophilic syndrome; PB HE, peripheral blood hypereosinophilia.

### Eosinophil-driven burden

Eosinophils are polymorphonuclear cells derived from hematopoietic precursors [8,9] and act as crucial mediators in hypereosinophilic disorders. Their development is primarily regulated by interleukin IL-3, IL-5, and granulocyte/macrophage colony-stimulating factor (GM-CSF), which promote their differentiation, survival and activation [3^▪^,^8^,^9^,^10^^▪▪^].

#### Activation and migration

In response to inflammatory signals such as IL-4, IL-13, and TNF-α, various cell types produce eotaxins-particularly eotaxin-1 (CCL11) which recruit eosinophils to tissues [8,9,10^▪▪^]. Upon reaching inflamed sites, eosinophils become overactivated and accumulate, initiating inflammatory responses that can lead to organ damage [3^▪^,^4^,^6^,^7^^▪▪^].

#### Granule proteins and cytotoxicity

Activated eosinophils release diverse mediators stored in their cytoplasmic granules, including major basic proteins (MBP1 and MBP2), eosinophil peroxidase (EPO), eosinophil cationic protein (ECP) and eosinophil-derived neurotoxin (EDN) [8,9,10^▪▪^]. These granule proteins exert cytotoxic effects, contributing to tissue injury and local inflammation.

#### Systemic effects

The combined actions of eosinophil-derived mediators result in pathological outcomes, such as leukocyte recruitment, persistent inflammation, thrombosis and fibrosis [3^▪^,^6^,^7^^▪▪^,^8^]. These processes underlie tissue damage and organ dysfunction characteristic of hypereosinophilic disorders.

### Clinical manifestations of hypereosinophilia syndrome

Hypereosinophilia syndrome (HES) presents a diverse array of clinical manifestations, affecting multiple organ systems with a spectrum ranging from asymptomatic to life-threatening complications. [2^▪▪^,^8^,^11^] (Fig. 1). The onset may be insidious, initially marked by asymptomatic eosinophilia, which can progress over years to symptomatic disease due to eosinophilic tissue infiltration and the deposition of eosinophil granules and proteins. In rarer cases, HES may have an acute onset [8,11,12]

### Initial clinical presentation and constitutional symptoms

Patients often report constitutional symptoms such as fatigue, recurrent fever, malaise and myalgia [3^▪^,^11^]. Regardless of the etiology, all patients may experience B-symptoms (weight loss, low-grade fever and night sweats) [5]. In a retrospective analysis involving 188 HES patients, dermatological features were the initial presentation in 37% of cases, followed by pulmonary (25%) and gastrointestinal (14%) involvement. Approximately 5% of patients presented with cardiac symptoms, while 6% were asymptomatic, with HES diagnosed incidentally [14].

### Organ-specific manifestations

#### Skin involvement

Skin manifestations are the most common, occurring in 69% of patients [14]. Symptoms include pruritus, urticarial lesions, and eczematous or papulonodular lesions, often seen in the lymphocytic variant of HES [15–17]. More severe presentations may include facial angioedema [18] palpable purpura, and mucosal ulcerations [19], especially in myeloid HES (M-HES), which may mimic Behçet's syndrome [1^▪^,^15^,^20^,^21^].

#### Respiratory manifestations

Pulmonary involvement occurs in 44% of patients [14], displaying a range of issues from rhinosinusitis and asthma to eosinophilic pneumonia. When eosinophilia extends to lung interstitium, it can lead to hypoxemia [1^▪^,^11^,^22^] and eosinophilic pleural effusions, which may cause lung compression [23].

#### Gastrointestinal involvement

Gastrointestinal symptoms affect 38% of patients [14] and can occur in isolation or conjunction with other organ dysfunctions. Manifestations may include eosinophilic colitis or enteritis, with symptoms such as abdominal pain, dyspepsia, and weight loss, while upper gastrointestinal issues may arise from eosinophilic esophagitis or gastritis [1^▪^].

#### Cardiac complications

Cardiac involvement may initially be asymptomatic but is associated with significant morbidity and mortality [1^▪^,^11^,^20^]. Cardiac manifestations include restrictive cardiomyopathy, heart failure, and thrombotic events due to eosinophilic infiltration, which promotes a pro-coagulant state [24,25].

#### Neurological manifestations

HES can lead to various neurological issues, including stroke [26] and peripheral neuropathy. A review identified cerebrovascular disease and altered mental status as the predominant CNS manifestations [27].

#### Hematologic findings

HES is characterized by eosinophilia, often accompanied by leukocytosis and other hematologic abnormalities such as anemia and thrombocytopenia [20].

#### Other manifestations

Rheumatological symptoms, including arthritis and myalgia [11,20], as well as ocular involvement and renal manifestations, may also be present, contributing to the clinical complexity of HES.

In summary, the clinical presentation of HES is multifaceted, impacting multiple systems and necessitating comprehensive evaluation and management to address the diverse range of symptoms and prevent severe complications (Table 1).

## CLASSIFICATION OF HYPEREOSINOPHILIA

Over the years, various classifications of eosinophil disorders have been proposed [1^▪^], typically distinguishing between primary (clonal), secondary (reactive), and idiopathic forms when the underlying causes remain elusive.

In 2023, Valent categorized hypereosinophilia into four main types based on etiology and clinical characteristics [3^▪^]:

(1) Hereditary (familial) HE (HEf): a rare genetically inherited form linked to mutations in genes on chromosome 5q31-33.
(2) Eosinophilia of unknown significance (HEus): a benign form of prolonged hypereosinophilia without signs of end-organ damage.
(3) Reactive HE (HEr): caused by external triggers like infections, allergies, or inflammation, where eosinophil levels rise as part of a systemic response.
(4) Neoplastic (clonal) HE (Hen): associated with hematologic malignancies such as myeloproliferative disorders or clonal expansions of activated T-lymphocytes.

Valent also classified HES and related disorders into [3^▪^]:

(1) Familial HES (HESFA): a rare hereditary variant without signs of cancer, where organ damage is directly caused by elevated eosinophils, excluding other immunological diseases.
(2) Idiopathic HES (HESI): a form with an unknown cause where high eosinophil levels cause organ damage without indicating cancer.
(3) Primary (neoplastic) HES (HESN): caused by blood cell cancers or related disorders, often involving mutations like PDGFRA, PDGFRB, and FGFR1, leading to organ damage.
(4) Secondary (reactive) HES (HESR): eosinophils increase as a response to another disease, often driven by cytokines, and contribute to organ damage.
(5) Special types of reactive HES:
  (a) *Lymphoid variant (L-HES):* clonal abnormal T cells drive eosinophil increase and organ damage.
  (b) *Specific syndromes:*
    (i) Gleich syndrome: features episodes of swelling (angioedema) with elevated eosinophils and abnormal T cells.
    (ii) Eosinophilic granulomatosis with polyangiitis (eGPA/Churg-Strauss): involves blood vessel inflammation, asthma, and lung issues, with some patients testing positive for ANCA antibodies.
    (iii) Eosinophilia myalgia syndrome (EMS): characterized by muscle pain, skin rash, breathing issues, and fatigue.
    (iv) IgG4-related disease (IgG4-RD): defined by increased immunoglobulin 4 (IgG4) antibodies, with around 30% of patients showing HES-like symptoms and organ damage.

Recently, the società italiana di allergologia asma e immunologia clinica (SIAAIC) task force proposed a revised classification where introduce several important conceptual distinctions [1^▪^] (Table 2).

(1) Clear separation between HE and HES: SIAAIC maintains the etiologic classification of HE (hereditary, reactive, clonal, idiopathic), but it explicitly distinguishes it from HES, which requires both eosinophilia and end-organ damage.
(2) Overlap syndromes are excluded from HES: diseases such as EGPA, ABPA, and IgG4-RD, which involve eosinophilia but are driven by broader immunologic mechanisms, are considered overlap forms of HE rather than subtypes of HES. This contrasts with Valent, who includes them under reactive HES.
(3) Lymphocytic variant treated as separate category: unlike Valent's inclusion of L-HES under reactive HES, SIAAIC recognizes lymphocytic HE as a distinct entity due to its clonal nature and potential for neoplastic transformation, representing a gray zone between reactive and neoplastic HE.
(4) Introduction of single-organ HE: SIAAIC adds a category for patients with eosinophilia confined to one organ, without systemic involvement, which is not explicitly addressed in Valent's model.

**Table 2:** Comparative table between valent and SIAAIC classification (HES subtypes)

Organ system	Frequency	Severity
Consistutional symptoms	Common (fatigue, fever, malaise, myalgia)	Varies; mild to moderate
Dermatologic	69%∗	Mild to severe
Respiratory	44%∗	Moderate to severe
Gastrointestinal	38%∗	Mild to moderate
Cardiac	20%∗	Severe, associated with high morbidity
Neurologic	∼20%∗	Moderate to severe
Haematologic	Common	Mild to severe
Ohters	Varied	Mild to moderate
Asymptomatic	Varied∗∗	–

HES, hypereosinophilic syndrome.

Basically in this revised classification HES subtypes include [1^▪^]:

(1) Myeloproliferative forms.
(2) Lymphocytic forms.
(3) HE with single organ involvement.
(4) Familial (hereditary) HE.
(5) Idiopathic HE.

This classification system enhances the understanding of hypereosinophilia, guiding diagnosis and potential treatment strategies [1^▪^].

A crucial differentiation within hypereosinophilia and HES lies between the lymphocytic and myeloid variants, which refer to different pathophysiologic mechanisms, clinical presentations, and treatment approaches (Table 3).

**Table 3:** Frequency and severity of manifestations by organ system.

Reference	HES subtypes
Valent *et al.*[3^▪^], 2023	Familial HES (HES_FA_) Idiopathic HES (HES_I_) Primary (neoplastic) HES (HES_N_) Secondary (reactive) HES (HES_R_) Special variants of HES_R_ Lymphoid variant of HES (L-HES) Defined syndromes (Gleich Syndrome, EGPA, EMS, IgG4-RD)
SIAAIC task force, Caminati *et al.*[1^▪^], 2024	Myeloproliferative forms. Lymphocytic forms. HE with single organ involvement. Familial (Hereditary) HE. Idiopathic HE.

HES, hypereosinophilic syndrome; IL, interleukin. (∗) The percentages are based on a retrospective analysis of 188 patients [14]. (∗∗) 6% of patients of a retrospective analysis present HE as an asymptomatic incidental finding; several of them develop clinical manifestations over the years [14]

The lymphocytic variant of hypereosinophilia (L-HES) is driven by clonal T-cell populations that produce cytokines (especially IL-5) to stimulate eosinophil proliferation. These T-cells are typically abnormal and the expansion of eosinophils is due to a T-cell-driven immune dysregulation rather than a primary eosinophil disorder.

(1) Clinical phenotypes: patients with L-HES often present with systemic symptoms such as fever, weight loss, fatigue, and organ infiltration by eosinophils, particularly in the lungs, skin, and gastrointestinal tract. Eosinophils may be elevated in peripheral blood, but the condition may also present with lymphadenopathy and splenomegaly due to the T-cell involvement. T-cell clonality and persistent eosinophilia suggest potential for progressive disease. Moreover these patients may have a history of atopic conditions or allergic diseases, which can overlap with autoimmune syndromes.
(2) Treatment implications: corticosteroids (e.g., prednisone) are often used as a first-line therapy to suppress eosinophil proliferation and T-cell activation. Immunosuppressive agents, such as methotrexate or cyclophosphamide may be required in refractory cases or when there is a high risk of organ damage due to chronic inflammation. Targeted therapies like monoclonal antibodies against IL-5 (e.g., mepolizumab) have been used to block eosinophil survival and reduce inflammation. In some cases, chemotherapy or T-cell-targeted treatments may be indicated for high-risk or aggressive cases.
  Instead the myeloid variants of HE (M-HES) refers to neoplastic or clonal eosinophilia originating from ∗myeloid progenitor cells. This form is primarily driven by genetic mutations that cause abnormal eosinophil proliferation, such as mutations in PDGFRA, PDGFRB, or FGFR1, leading to myeloproliferative diseases.
(3) Clinical phenotype: patients with M-HES often present with eosinophil-driven organ damage, including involvement of the heart, lung, liver, and skin. These patients may show symptoms related to eosinophil infiltration, such as cardiomyopathy, pulmonary fibrosis, skin rashes, and gastrointestinal complications. Unlike lymphocytic HES, myeloid HES tends to be associated with clonal eosinophil expansion, and genetic testing often reveals chromosomal translocations or point mutations that drive eosinophil dysregulation.
(4) Treatment implications: Tyrosine kinase inhibitors (e.g., imatinib) are the cornerstone of therapy for patients with PDGFRA-related myeloid HES or FGFR1 rearrangements, as they target the specific genetic abnormalities driving eosinophil proliferation. In the absence of these mutations, patients may be treated with chemotherapy, interferon-alpha, or biological agents (e.g., mepolizumab) to control eosinophilia. Finally stem cell transplant may be an option for patients with refractory or aggressive disease.

In conclusion, the categorization of hypereosinophilia and hypereosinophilic syndromes is still developing, and the important differences between lymphocytic and myeloid variations aid in directing diagnostic and therapeutic approaches. By comprehending these variations and how they connect to the underlying pathophysiology, clinicians may better customize interventions to meet patients’ individual needs.

## DIAGNOSIS OF HYPEREOSINOPHILIA

The clinical manifestations of HES are often heterogeneous and nonspecific, presenting significant challenges in diagnosis [11]. Thus, a comprehensive medical history, physical examination, laboratory tests and imaging studies are vital to identify the underlying etiology and assess potential organ involvement, which may remain asymptomatic in the early stages of the disease [1^▪^,3^▪^,^5^,^11^].

### Step 1: confirm hypereosinophilia

The initial diagnostic step involves confirming the laboratory findings that demonstrate an elevated AEC. This requires two repeated blood measurements, taken at least two weeks apart, to establish persistent eosinophilia [1^▪^,3^▪^].

### Step 2: exclude secondary forms

The diagnosis must include excluding rare familial forms of hypereosinophilia by obtaining a detailed medical and family history. It is also important to assess for predispositions to neoplastic diseases, including T-cell lymphomas, Hodgkin lymphoma, and acute lymphoblastic leukemia [1^▪^,3^▪^]. A thorough medical and pharmacological history is critical to identify any concurrent conditions or medications that may provoke HES. Laboratory tests conducted should include complete blood count, C-reactive protein, serum protein electrophoresis, renal function tests, liver function tests, immunoglobulins, an autoimmune panel and complement levels [1^▪^,3^▪^,^11^]. Consideration of parasitic infections is essential; thus, a comprehensive evaluation of the patient's travel history and lifestyle, coupled with testing for Strongyloides IgG, ova and parasite testing and stool cultures, should be undertaken [1^▪^,^2^^▪▪^,^5^].

### Step 3: exclude clonal forms

In the absence of reactive causes, primary clonal hypereosinophilia should be investigated through blood count, bone marrow studies, and cytogenetic/molecular testing (karyotyping, FISH, flow cytometry, RT-PCR, NGS) [1^▪^,^2^^▪▪^,3^▪^,^5^]. These detect key tyrosine kinase gene rearrangements -including FIP1L1::PDGFRA, PDGFRB, FGFR1, JAK2, and ABL1, as well as other relevant mutations [1^▪^,^2^^▪▪^]. RT-PCR for FIP1L1::PDGFRA is often preferred in clinical practice due to its high sensitivity [1^▪^]. Clonality is suggested by cytopenias, thrombocytosis, polycythemia, monocytosis, basophilia and elevated vitamin B12 and tryptase levels [1^▪^,^11^,^29^]. Final diagnosis should follow WHO (ICC) and ICOG-EO criteria, integrating all clinical, morphological and molecular data to guide classification and targeted therapy [3^▪^]. Ultimately, the presence or absence of an associated HES should be addressed [3^▪^].

### Step 4: diagnosis by exclusion of idiopathic hypereosinophilia

If neither primary nor secondary causes of hypereosinophilia can be identified, the diagnosis of idiopathic hypereosinophilia (iHE) or idiopathic HES (iHES) is established [5].

### Step 5: evaluate organ involvement

Regardless of the presence of symptoms, the evaluation of chronic hypereosinophilia must include both histological examination and imaging studies to assess potential organ involvement. For cardiac assessment at diagnosis, serum troponin T and NT-proBNP levels should be measured, alongside an electrocardiogram (ECG) and echocardiography [1^▪^,^11^,^24^]. Cardiac MRI (CMR) serves as a second-line test, offering higher sensitivity for detecting ventricular thrombi, while myocardial biopsy remains the gold standard for assessing cardiac damage associated with HES [1^▪^,^30^]. For pulmonary evaluation, function tests including diffusion capacity for carbon monoxide (DLCO), chest X-ray, and high-resolution computed tomography (HRCT) are essential at baseline and during follow-up. Lung biopsy and bronchoalveolar lavage fluid (BALF) analysis may help detect eosinophilic infiltration in the airways and lung tissues [1^▪^]. In cases presenting gastrointestinal symptoms or abnormalities in liver enzymes, endoscopic examinations of the upper and lower gastrointestinal tract may aid in diagnosis, although sensitivity can be limited [31]. Histopathological confirmation should include biopsies from both normal and affected mucosa to enhance diagnostic accuracy, with a minimum of six specimens recommended for thorough evaluation [3^▪^].

## CURRENT THERAPIES AND FUTURE PERSPECTIVES

In the absence of organ dysfunction, there is currently insufficient evidence to warrant initiating treatment based solely on elevated eosinophil counts. However, some specialists propose that treatment may begin at an AEC of 1.5–2 × 10^9^/l. The therapeutic approach should be tailored according to the specific etiological classification of HE or HES and the presence or absence of specific mutations.

### Targeted therapies for myeloid hypereosinophilic syndrome

In patients with M-HES, particularly those harboring the FIP1L1::PDGFRA fusion gene, imatinib represents a cornerstone of therapy [32,33]. Even at low doses (100 mg/day), imatinib induces rapid and sustained hematologic, cytogenetic, and often molecular remission. Its efficacy has dramatically altered the prognosis of FIP1L1–PDGFRA–positive HES, with long-term disease control and steroid independence achievable in most cases. For patients with MLN-eo-TK and PDGFRB fusions, a daily dose of 400 mg is recommended, reducing to 100 mg during maintenance [2^▪▪^]. However, resistance to imatinib can occur, particularly in the presence of atypical or variant PDGFRA mutations, or in patients with other kinase-driven clonal disorders (e.g., PDGFRB, FGFR1, or JAK2 fusions). In these cases, alternative tyrosine kinase inhibitors (TKIs) such as dasatinib or nilotinib may be attempted, although evidence remains limited [2^▪▪^]. However, criteria for HSCT remain poorly defined within current guidelines [1^▪^]. For patients with persistent disease despite TKI therapy, or in the presence of additional high-risk mutations (e.g., ASXL1, RUNX1), hematopoietic stem cell transplantation (HSCT) may be considered, particularly in younger, fit patients with progressive disease or marrow failure. [34]. Pemigatinib has recently been approved for relapsed or refractory diseases associated with FGFR1 rearrangements [35]. Ongoing clinical trials are evaluating the efficacy of ruxolitinib, a JAK1/JAK2 inhibitor, in JAK2-rearranged or mutated eosinophilic neoplasms *(clinicaltrials.gov NCT03801434 and NCT00044304)*. A diagnostic work-up that includes cytogenetics, FISH, RT-PCR, and next-generation sequencing is critical in identifying molecular drivers and guiding targeted therapy selection [2^▪▪^].

### Therapies for idiopathic hypereosinophilic syndrome

A stepwise treatment algorithm is commonly applied in clinical practice:

(1) First-line: Corticosteroids remain the first-line treatment in iHES, with prednisone typically initiated at a dose of 1 mg/kg/day, tapered per clinical and hematologic response [8]. However, many patients relapse during tapering or develop corticosteroid-related adverse effects, necessitating second-line therapies [2^▪▪^].
(2) Steroid-sparing agents: In cases of steroid-dependence, intolerance, or relapse, immunomodulatory or cytotoxic agents such as hydroxyurea or interferon-α are considered. The choice depends on disease severity, patient comorbidities, and response history [1^▪^].
(3) Biologic agents: Biologics are increasingly favored over cytoreductive therapies in relapsing or refractory HES due to their favorable safety profile. However, cytoreductive agents remain a valuable option in cases of rapidly progressive or organ-threatening disease, or when biologics are not accessible. [1^▪^].

#### Empirical antiparasitic therapy

The effectiveness of empirical antiparasitic therapy in unexplained chronic HE is debatable due to the lack of published evidence [1^▪^]. There is a clinical experience with cases where empirical antiparasitic treatment permitted otherwise inexplicable HE to completely and sustainably return to normal (despite negative well conducted parasite testing). However, for a number of reasons, it might be suitable in certain situations, such the available assays and the varying sensitivity of parasite serology for stool parasite identification, in addition to the medications favorable safety and affordability. Treatment with fenbendazole or albendazole or praziquantel is indicated for any eosinophilia <1.5× 10^9^/l in the absence of an obvious cause and contraindications [36].

### Biologics

Given that interleukin-5 (IL-5) is a major cytokine mediating eosinophil growth and activation, several studies have assessed the potential of IL-5-targeting biologics that have been demonstrated to be a safe and efficient substitute in idiopathic HES and lymphocytic HES, the latter characterized by overproduction of IL-5 by dysregulated T cells [37,38^▪▪^] Mepolizumab has been approved for HES treatment, showing effectiveness in reducing eosinophil counts and HES flare-ups [39]. Benralizumab 30 mg every 4 weeks showed to be noninferior to mepolizumab in adults with EGPA (but is not yet approved) [40]. A phase II study has demonstrated the efficacy of benralizumab in PDGFRA-negative HES [41]. A phase III, randomized, placebo-controlled study is presently being conducted to treat HES with benralizumab *(clinicaltrials.gov NATRON, NCT04191304)*. A randomized, double-blind, placebo-controlled trial is underway to evaluate the efficacy and safety of depemokimab in adults with HES (*clinicaltrials.gov DESTINY, NCT05334368*). With its improved binding affinity for interleukin-5, depemokimab is an ultra-long-acting biologic treatment that may allow for efficient 6-month dosing intervals. Effectiveness in treating patients with severe asthma and eosinophilic phenotype has already been demonstrated [42]. Additionally, dupilumab is a human monoclonal antibody against interleukin (IL)-4 receptor alpha that inhibits IL-4/IL-13 axis, which is responsible for several Th2-type inflammatory mechanisms including migration of eosinophils to tissues. Therefore is ongoing a study with dupilumab as add-on therapy for HES in patients with complete hematologic and partial clinical remission on eosinophil-lowering biologic agents (anti-IL-5/anti-IL5-Rα) with the aim of reduce residual pulmonary, skin, esophageal, and sinus symptoms (*clinicaltrial.gov NCT06477653*). Table 4

**Table 4:** Currently available and investigational biologic agents used in HES

Drug	Target	Indication	Status	Clinical trial
Mepolizumab	Anti–IL-5	iHES and L-HES	Approved (FDA, EMA)	NCT02836496
Benralizumab	Anti–IL-5Rα	PDGFRA-negative, refractory HES	Off-label / Under investigation	NATRON: NCT04191304
Depemokimab	Anti–IL-5 (long-acting)	HES with residual tissue symptoms	Phase III	DESTINY: NCT05334368
Dupilumab	Anti–IL-4Rα	HES with uncontrolled tissue symptoms	Investigational	NCT06477653

HES, hypereosinophilic syndrome; IL, interleukin.

### Emerging therapies and future directions

Novel biologics and small molecules targeting IL-33, thymic stromal lymphopoietin (TSLP), or Siglec-8 (*clinicaltrial.gov NCT04322708)* are under investigation in early-phase trials [12,41]. Future strategies include precision medicine approaches, better stratification tools, and multicenter registries to improve therapeutic outcomes [34].

### Long-term monitoring and follow-up

Long-term monitoring of patients with HES should be individualized and based on clinical phenotype, disease severity, organ involvement, and treatment response. Regular clinical evaluations are recommended, supported by targeted instrumental assessments (such as thoracic imaging, echocardiography, and pulmonary function tests) especially for previously affected organs. Laboratory follow-up should include eosinophil counts (AEC), total immunoglobulin E (IgE), ECP, vitamin B12, serum tryptase, and inflammatory markers like CRP and ESR. This personalized approach allows for timely adjustment of therapy and detection of disease progression or relapse [1^▪^]. Emerging biomarkers such as serum IL-5 and thymus and activation-regulated chemokine (TARC) are under evaluation for their potential role in monitoring disease activity and therapeutic response in HES [8,34]. Multidisciplinary follow-up (hematology, immunology, cardiology, dermatology) ensures comprehensive care [13,28].

## CONCLUSION

**FIGURE 2:**
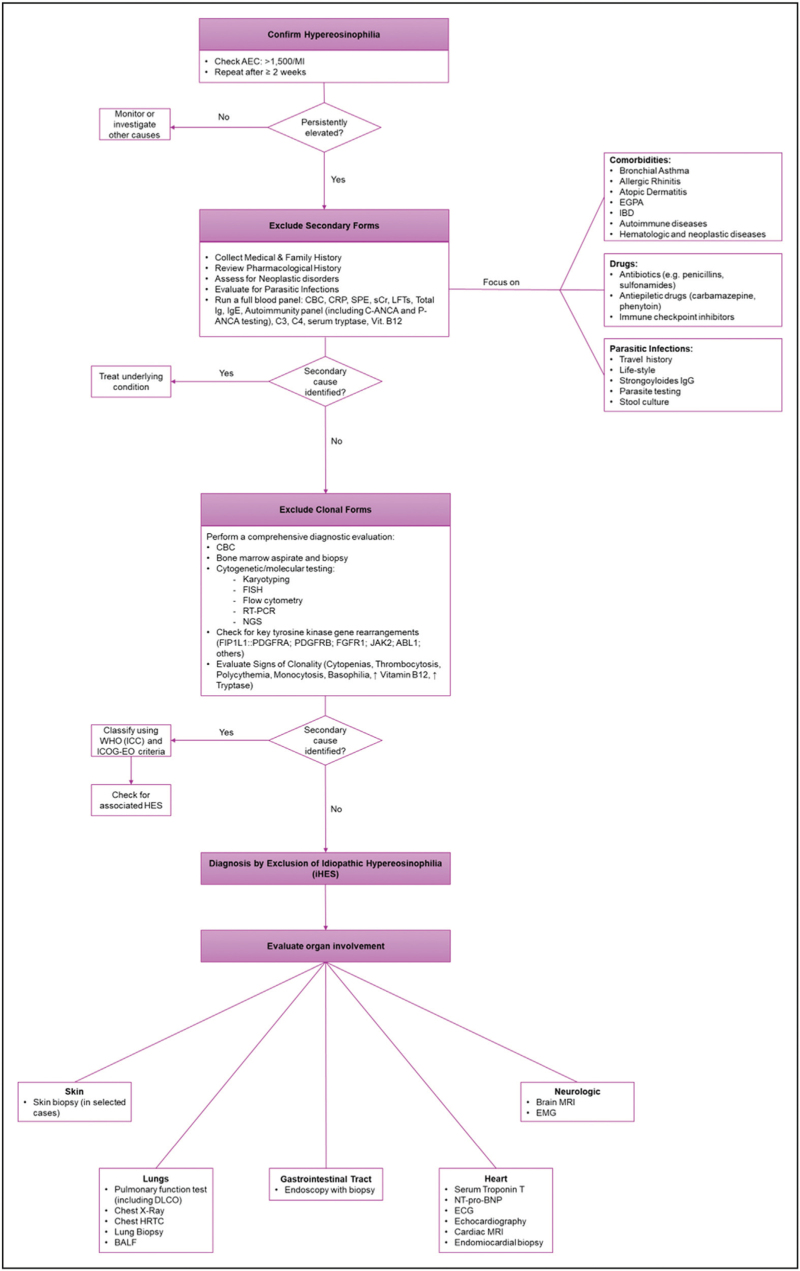
Diagnostic algorithm for HES. Imagine created by Biorender. BALF, bronchoalveolar lavage fluid; CBC, complete blood count; CRP, C-reactive protein; ECG, electrocardiogram; EGPA, eosinophilic granulomatosis with polyangiitis; EMG, electromyography; FISH, fluorescence in situ hybridization; HRCT, high-resolution computed tomography; IBD, inflammatory bowel disease; Ig, immunoglobulins; LFTs, liver function tests; MRI, magnetic resonance imaging; NGS, next-generation sequencing; RT-PCR, reverse transcription polymerase chain reaction; sCr, serum creatinine; SPE, serum protein electrophoresis.

Hypereosinophilic disorders comprise a heterogeneous group of conditions with a wide spectrum of clinical presentations and pathophysiological mechanisms (Fig. 2). A precise etiologic diagnosis is key to guiding treatment choices and preventing irreversible organ damage. While the development of targeted therapies has significantly improved outcomes in specific subsets, particularly in M-HES, many patients still rely on corticosteroids or off-label agents, underlining the limitations of current strategies. Biologics have emerged as a promising steroid-sparing option, especially in iHES and L-HES, but access and evidence remain variable across indications. Furthermore, real-world management requires dynamic monitoring of disease activity, integrating clinical, hematologic, and imaging parameters, alongside emerging biomarkers. Despite advances, major gaps remain in classification, treatment algorithms, and long-term follow-up practices. Future research should prioritize multicenter prospective registries, standardized response criteria, and precision medicine approaches to individualize care. A multidisciplinary framework is essential to address the complexity of hypereosinophilic syndromes and ensure optimal patient outcomes.

## Acknowledgements

*G.C., G.T., M.C., R.S.C. prepared the manuscript. All coauthors provided a critical review of the manuscript. All authors have read and agreed to the published version of the manuscript. Authorship must be limited to those who have contributed substantially to the work reported. We do not use generative AI tools, all the figures are originals.*

### Financial support and sponsorship

*None.*

### Conflicts of interest

*There are no conflicts of interest.*

## REFERENCES AND RECOMMENDED READING

Papers of particular interest, published within the annual period of review, have been highlighted as:

▪ of special interest
▪▪ of outstanding interest

## References

[1▪] Caminati M Brussino L Carlucci M. Managing patients with hypereosinophilic syndrome: a statement from the Italian Society of Allergy. Asthma Clin Immunol Cells 2024; 13:118. 10.3390/cells13141180 PMC11274683 39056762

[2▪▪] Shomali W Gotlib J. World Health Organization and International Consensus Classification of eosinophilic disorders: 2024 update on diagnosis, risk stratification, and management. Am J Hematol 2024; 99:946–968. 38551368 10.1002/ajh.27287

[3▪] Valent P Klion AD Roufosse F. Proposed refined diagnostic criteria and classification of eosinophil disorders and related syndromes. Allergy 2023; 78:47–59. 36207764 10.1111/all.15544 PMC9797433

[4] Valent P Klion AD Horny HP. Contemporary consensus proposal on criteria and classification of eosinophilic disorders and related syndromes. J Allergy Clin Immunol 2012; 130:607–612. e9. 22460074 10.1016/j.jaci.2012.02.019 PMC4091810

[5] Thomsen GN Christoffersen MN Lindegaard HM. The multidisciplinary approach to eosinophilia. Front Oncol 2023; 13:1193730. 37274287 10.3389/fonc.2023.1193730 PMC10232806

[6] Kita H. Eosinophils: multifaceted biological properties and roles in health and disease. Immunol Rev 2011; 242:161–177. 21682744 10.1111/j.1600-065X.2011.01026.x PMC3139217

[7▪▪] Weller PF Spencer LA. Functions of tissue-resident eosinophils. Nat Rev Immunol 2017; 17:746–760. 28891557 10.1038/nri.2017.95 PMC5783317

[8] Valent P Degenfeld-Schonburg L Sadovnik I. Eosinophils and eosinophil-associated disorders: immunological, clinical, and molecular complexity. Semin Immunopathol 2021; 43:423–438. 34052871 10.1007/s00281-021-00863-y PMC8164832

[9] Sanchez Santos A Socorro Avila I Galvan Fernandez H. Eosinophils: old cells, new directions. Front Med (Lausanne) 2025; 11:1470381. 39886455 10.3389/fmed.2024.1470381 PMC11780905

[10▪▪] Gigon L Fettrelet T Yousefi S. Eosinophils from A to Z. Allergy 2023; 78:1810–1846. 37102676 10.1111/all.15751

[11] Caminati M Carpagnano LF Alberti C. Idiopathic hypereosinophilic syndromes and rare dysimmune conditions associated with hyper-eosinophilia in practice: An innovative multidisciplinary approach. World Allergy Organi J 2024; 17:100928. 10.1016/j.waojou.2024.100928 PMC11327453 39156600

[12] Helbig G Klion AD. Hypereosinophilic syndromes – an enigmatic group of disorders with an intriguing clinical spectrum and challenging treatment. Blood Rev 2021; 49:100809. 33714638 10.1016/j.blre.2021.100809

[13] Klion AD. Approach to the patient with suspected hypereosinophilic syndrome. Hematology 2022; 2022:47–54. 36485140 10.1182/hematology.2022000367 PMC9821533

[14] Ogbogu PU Bochner BS Butterfield JH. Hypereosinophilic syndrome: a multicenter,;1; retrospective analysis of clinical characteristics and response to therapy. J Allergy Clin Immunol 2009; 124:1319–1325. e3. 19910029 10.1016/j.jaci.2009.09.022 PMC2829669

[15] Requena G van den Bosch J Akuthota P. Clinical profile and treatment in hypereosinophilic syndrome variants: a pragmatic review. J Allergy Clin Immunol Pract 2022; 10:2125–2134. 35470096 10.1016/j.jaip.2022.03.034

[16] Lefèvre G Copin MC Staumont-Sallé D. The lymphoid variant of hypereosinophilic syndrome. Medicine 2014; 93:255–266. 25398061 10.1097/MD.0000000000000088 PMC4602413

[17] Simon HU Plötz SG Dummer R Blaser K. Abnormal clones of T cells producing interleukin-5 in idiopathic eosinophilia. N Engl J Med 1999; 341:1112–1120. 10511609 10.1056/NEJM199910073411503

[18] Radonjic-Hoesli S Brüggen MC Feldmeyer L. Eosinophils in skin diseases. Semin Immunopathol 2021; 43:393–409. 34097126 10.1007/s00281-021-00868-7 PMC8241748

[19] Hayashi M Kawaguchi M Mitsuhashi Y Suzuki T. Case of hypereosinophilic syndrome with cutaneous necrotizing vasculitis. J Dermatol 2008; 35:229–233. 18419681 10.1111/j.1346-8138.2008.00450.x

[20] Curtis C Ogbogu P. Hypereosinophilic syndrome. Clin Rev Allergy Immunol 2016; 50:240–251. 26475367 10.1007/s12016-015-8506-7

[21] Leiferman KM Gleich GJ Peters MS. Dermatologic manifestations of the hypereosinophilic syndromes. Immunol Allergy Clin North Am 2007; 27:415–441. 17868857 10.1016/j.iac.2007.07.009

[22] Bay P Groh M Gaillet A. Extracorporeal membrane oxygenation for refractory acute eosinophilic pneumonia. J Crit Care 2024; 79:154437. 37782978 10.1016/j.jcrc.2023.154437

[23] Soma S. Pericardial effusion and progressive bilateral effusion as rare presentations of idiopathic hypereosinophilic syndrome. Cureus 2023; 15:e44495. 37791155 10.7759/cureus.44495 PMC10544721

[24] Bondue A Carpentier C Roufosse F. Hypereosinophilic syndrome: considerations for the cardiologist. Heart 2022; 108:164–171. 34172539 10.1136/heartjnl-2020-317202

[25] Réau V Vallée A Terrier B. Venous thrombosis and predictors of relapse in eosinophil-related diseases. Sci Rep 2021; 11:6388. 33737704 10.1038/s41598-021-85852-9 PMC7973521

[26] Ono R Iwahana T Kato H. Literature reviews of stroke with hypereosinophilic syndrome. Int J Cardiol Heart Vasc 2021; 37:100915. 34888412 10.1016/j.ijcha.2021.100915 PMC8636825

[27] Lee D Ahn TB. Central nervous system involvement of hypereosinophilic syndrome: a report of 10 cases and a literature review. J Neurol Sci 2014; 347 (1–2):281–287. 25455301 10.1016/j.jns.2014.10.023

[28] Leru PM. Eosinophilic disorders: evaluation of current classification and diagnostic criteria, proposal of a practical diagnostic algorithm. Clin Transl Allergy 2019; 9:36. 31367340 10.1186/s13601-019-0277-4 PMC6657042

[29] Mattis DM Wang SA Lu CM. Contemporary classification and diagnostic evaluation of hypereosinophilia. Am J Clin Pathol 2020; 154:305–318. 32525541 10.1093/ajcp/aqaa056

[30] Ogbogu PU Rosing DR Horne MK. Cardiovascular manifestations of hypereosinophilic syndromes. Immunol Allergy Clin North Am 2007; 27:457–475. 17868859 10.1016/j.iac.2007.07.001 PMC2048688

[31] Kuang FL Curtin BF Alao H. Single-organ and multisystem hypereosinophilic syndrome patients with gastrointestinal manifestations share common characteristics. J Allergy Clin Immunol Pract 2020; 8:2718–2726. e2. 32344186 10.1016/j.jaip.2020.04.025 PMC7483350

[32] David M Cross NCP Burgstaller S. Durable responses to imatinib in patients with PDGFRB fusion gene–positive and BCR-ABL–negative chronic myeloproliferative disorders. Blood 2007; 109:61–64. 16960151 10.1182/blood-2006-05-024828

[33] Metzgeroth G Schwaab J Gosenca D. Long-term follow-up of treatment with imatinib in eosinophilia-associated myeloid/lymphoid neoplasms with PDGFR rearrangements in blast phase. Leukemia 2013; 27:2254–2256. 23615556 10.1038/leu.2013.129

[34] Lefèvre G Bleuse S Puyade M. Hypereosinophilia and hypereosinophilic syndromes: first findings from a nationwide multicenter cohort. Allergy 2025; 80:1100–1110. 39757773 10.1111/all.16463

[35] Verstovsek S Gotlib J Vannucchi AM. FIGHT-203, an ongoing phase 2 study of pemigatinib in patients with myeloid/lymphoid neoplasms (MLNs) with fibroblast growth factor receptor 1 (*FGFR1*) rearrangement (MLN *FGFR1*): a focus on centrally reviewed clinical and cytogenetic responses in previously treated patients. Blood 2022; 140: (Suppl 1): 3980–3982.

[36] Groh M Rohmer J Etienne N. French guidelines for the etiological workup of eosinophilia and the management of hypereosinophilic syndromes. Orphanet J Rare Dis 2023; 18:100. 37122022 10.1186/s13023-023-02696-4 PMC10148979

[37] Lombardi C Comberiati P Ridolo E. Anti-IL-5 pathway agents in eosinophilic-associated disorders across the lifespan. Drugs 2024; 84:661–684. 38849701 10.1007/s40265-024-02037-0 PMC11196311

[38▪▪] Jain P Rowell J Edmonds C. A systematic literature review of real-world outcomes following anti-interleukin-5 and anti-interleukin-5 receptor alpha treatment for hypereosinophilic syndrome: case reports and cohort studies. Blood 2024; 144: (Suppl 1): 5090–15090.

[39] Pavord ID Bel EH Bourdin A. From DREAM to REALITI-A and beyond: mepolizumab for the treatment of eosinophil-driven diseases. Allergy 2022; 77:778–797. 34402066 10.1111/all.15056 PMC9293125

[40] Veltman Y Aalbers AM Hermans MAW Mutsaers PGNJ. Single-center off-label benralizumab use for refractory hypereosinophilic syndrome demonstrates satisfactory safety and efficacy. EJHaem 2025; 6:e1014. 39866927 10.1002/jha2.1014 PMC11756971

[41] Kuang FL Legrand F Makiya M. Benralizumab for *PDGFRA*-negative hypereosinophilic syndrome. N Engl J Med 2019; 380:1336–1346. 30943337 10.1056/NEJMoa1812185 PMC6557265

[42] Jackson DJ Wechsler ME Jackson DJ. Twice-yearly depemokimab in severe asthma with an eosinophilic phenotype. N Engl J Med 2024; 391:2337–2349. 39248309 10.1056/NEJMoa2406673

